# Complete Sequence of a F33:A-:B- Conjugative Plasmid Carrying the *oqxAB*, *fosA3*, and *bla*_CTX-M-55_ Elements from a Foodborne *Escherichia coli* Strain

**DOI:** 10.3389/fmicb.2016.01729

**Published:** 2016-10-27

**Authors:** Marcus H. Wong, Miaomiao Xie, Liqi Xie, Dachuan Lin, Ruichao Li, Yuanjie Zhou, Edward W. Chan, Sheng Chen

**Affiliations:** ^1^Shenzhen Key Laboratory for Food Biological Safety Control, Food Safety and Technology Research Centre, Shenzhen Research Institute, The Hong Kong Polytechnic UniversityShenzhen, China; ^2^State Key Laboratory of Chirosciences, Department of Applied Biology and Chemical Technology, The Hong Kong Polytechnic UniversityKowloon, Hong Kong; ^3^Division of Food Inspection and Supervision, Shenzhen Entry-Exit Inspection and Quarantine BureauShenzhen, China

**Keywords:** IncFII plasmids, *oqxAB*, *E. coli*, *fosA3*, dissemination

## Abstract

This study reports the complete sequence of pE80, a conjugative IncFII plasmid recovered from an *Escherichia coli* strain isolated from chicken meat. This plasmid harbors multiple resistance determinants including *oqxAB*, *fosA3*, *bla*_CTX-M-55,_ and *bla*_TEM-1_, and is a close variant of the recently reported p42-2 element, which was recovered from *E. coli* of veterinary source. Recovery of pE80 constitutes evidence that evolution or genetic re-arrangement of IncFII type plasmids residing in animal-borne organisms is an active event, which involves acquisition and integration of foreign resistance elements into the plasmid backbone. Dissemination of these plasmids may further compromise the effectiveness of current antimicrobial strategies.

## Introduction

The increasing prevalence of bacterial strains producing CTX-M-type extended-spectrum β-lactamases (ESBLs) in clinical setting poses a serious public health threat worldwide (Ho et al., 2012). In recent years, fosfomycin has been increasingly used to treat both urinary and systemic infections due to rapid dissemination of multidrug-resistant Gram-negative bacteria, especially strains of carbapenem-resistant *Enterobacteriaceae*, in clinical settings (Falagas et al., 2010). However, increased clinical usage of fosfomycin rapidly resulted in selection of the corresponding resistance gene, namely *fosA3*, in clinical *Escherichia coli* and *Klebsiella pneumoniae* isolates (Wachino et al., 2010; Lee et al., 2012; Ho et al., 2013a,b; Sato et al., 2013). The *fosA3* gene has been consistently found to be located in IncFII type plasmids, and often detectable in CTX-M-producing and multidrug-resistant *E. coli* strains recoverable from animals as well as patients in China, Japan, and Korea (Wachino et al., 2010; Lee et al., 2012; Ho et al., 2013a,b; Sato et al., 2013). One of the representative multi-resistance IncFII type plasmid is pHN7A8. It was harbored by an *E. coli* of animal origin, and encoded *fosA3*, *bla*_CTX-M-55,_ and *rmtB* (He et al., 2013). On the other hand, an increasing number of antibiotic resistance determinants are known to be harbored by IncFII plasmids, including one of the major plasmid-mediated quinolone resistance determinants, the Resistance-Nodulation-Division (RND) eﬄux pump gene, *oqxAB*, which has become frequently detectable in fluoroquinolone-resistant *E. coli* and *Salmonella* strains. Except for the original *oqxAB*-bearing plasmid, pOLA52, which was first recovered in *E. coli*, information regarding the mobile elements that mediate the transmission of *oqxAB* among strains of the *Enterobacteriaceae* family remains scarce. Recently, the complete sequence of an IncFII plasmid carrying *oqxAB*, *fosA3*, *bla*_CTX-M-55,_ and *floR* from an *E. coli* of veterinary origin has been described (Yang et al., 2016). In this study, we reported the complete sequence of a F33:A-:B- conjugative plasmid carrying the *oqxAB*, *fosA3*, *floR*, and *bla*_CTX-M-55_ genes harbored by an *E. coli* strain isolated from chicken meat from a retail store in Shenzhen, China. Although the widespread dissemination of identical or highly similar plasmids, which can confer resistance to several important classes of antibiotics in clinical strains remained to be seen, it will undoubtedly pose an enormous public health threat. Intense research efforts are therefore warranted to unveil the genetic features of this new plasmid type in order to provide essential information required to devise new strategies to prevent further dissemination of such multidrug resistance-encoding determinants among human pathogens.

## Materials and Methods

### Bacterial Isolation

Fresh chicken meat samples were purchased from wet markets and supermarkets at Shenzhen, People’s Republic of China during 2010–2013. Food samples collected were transported on ice to the laboratory for isolation within 6 h. Twenty-five grams of chicken sample were placed in 100-mL Buffered Peptone Water (BPW; Difco, Detroit, MI, USA) and was incubated at 35°C for 24 h. A loopful of enriched homogenate was streaked on MacConkey agar plate and incubated at 35°C for 24 h. Suspected colonies were purified and further identified by API20E (BioMérieux).

### Antimicrobial Susceptibility Testing

*Escherichia coli* E80 and its transconjugant were subjected to antimicrobial susceptibility testing using the agar-dilution method, and the results were interpreted according to the CLSI guidelines (CLSI, 2015). Briefly, bacterial cultures were grown overnight on Mueller Hinton agar (MHA) and bacterial suspension of 0.5 McFarland standard were prepared, and were then inoculated on a series of MHA containing different concentrations of antimicrobials. The plates were incubated at 37°C for 24 h. Minimal inhibitory concentration (MIC) was determined at the antimicrobial concentration that inhibit bacterial growth. Eleven antimicrobials were tested: ampicillin, ceftriaxone, cefotaxime, kanamycin, nalidixic acid, ciprofloxacin, tetracycline, sulfamethoxazale, chloramphenicol, fosfomycin, and olaquindox. *E. coli* strains ATCC 25922 and *Staphylococcus aureus* strain ATCC 29213 were used as quality control.

### Resistance Genes Detection and Conjugation Experiments

Resistance genes harbored by E80 were screened by PCR using the primers listed in **Table 1**. A conjugative experiment was carried out as previously described (Jacoby et al., 2003) using sodium azide-resistant *E. coli* J53 strain as recipient. Briefly, overnight culture of E80 and recipient strains J53 were mixed and collected on a filter, which was subjected to overnight incubation on a blood agar plate. The mixture was then spread on double selective blood agar plates containing olaquindox (32 μg/mL) and sodium azide (100 μg/mL) to select drug resistant transconjugants.

**Table 1:** Primers used in this study.

Primer	Sequence (5′–3′)	Purpose
*bla*_TEM_	F: GAGAGTTTTCGCCCCGAAGA;	Detection of
	R: CGTTCATCCATAGTTGCCTGAC	Resistance Gene
*bla*_CTX-M-1_ Group	F: TTAGGAARTGTGCCGCTGYA;
	R: CGATATCGTTGGTGGTRCCAT	
FosA3	F: GCGTCAAGCCTGGCATTT;
	R: GCCGTCAGGGTCGAGAAA	
OqxA	F: GCGTCTCGGGATACATTGAT;
	R: GGCGAGGTTTTGATAGTGGA	
repN	F: GTCTAACGAGCTTACCGAAG	Screening of pE80
	R: ACGGTCATTTAACCAAGCATG
repFII	F: CTGATCGTTTAAGGAATTTT;
	R: CACACCATCCTGCACTTA	
pemKI	F: ACAGACGCCCGCAATATTCA;
	R: TGATAGTCTCCGGAACCCGT	
vagD	F: ATCATGCGTGAACAGCCAGA;
	R: ATCTTCGTGGTGGCGTCTAC	
ISPa38-oqxA	F: GCGTCGAGCACGTCC
	TTCGCGGCGGGAGCGGCCCG;
	R: CGCGGTCAGGTGAATGTTTC	
Ars-RepA	F: CCGCTGGCAAAAATGGGTAA;
	R: GAAGCTGTGAAATACGTCTG	
mob	F: GGAGAAGCTGAATGCACTGG;
	R: GCTTTCACGACCTGATTAAG	
IS26-CTXM1	F: AGCGCTCATCAGCACGATAA;
	R: GGATAATCAACGCCACGCTG	
TEM-orf477	F: TCTAGCTTCCCGGCAACAAT;
	R: GGCAACAGTAGCTCGAAGGT	
TraX-finO	F: CCATTCTTCTGATGGCGCTG;
	R: CGTCACATACCCTTCCGTGT	

### Plasmid Sequencing and Analysis

The plasmid pE80 was extracted from the transconjugant using the Qiagen Plasmid Midi-Prep Kit, followed by plasmid sequencing using the PacBio RSII Single Molecular Real-time (SMRT) Sequencer at the Wuhan Institute of Biotechnology, People’s Republic of China. Reads were assembled by Hierarchical Genome Assembly Process (HGAP) of the SMRT Analysis software (Chin et al., 2013). No gaps were formed after sequencing. Plasmid sequence was annotated by RAST followed by manual review, using BLASTP. Sequence comparisons and alignment were performed by the BLASTN and EasyFig (Sullivan et al., 2011). Plasmid Multilocus Sequence Typing (pMLST) was performed on pE80 by extracting corresponding sequence using the IncF type primer sets and compared with the database (Villa et al., 2010).

### PCR Screening of pE80

Ten sets of primers targeting both conserved and multi-drug resistance regions of pE80 were adopted in PCR screening in order to determine the dissemination of this plasmid among *E. coli* isolates. The primers used are listed in **Table 1**. A total of 190 *E. coli* isolates were included in the screening. These isolates were isolated from meat products collected in Shenzhen, China during 2010–2012 as aforementioned.

## Results And Discussion

*Escherichia coli* strain E80 was isolated from a chicken meat sample purchased from a supermarket in Shenzhen, People’s Republic of China in 2013. Antimicrobial susceptibility tests showed that the isolate was resistant to ampicillin, ceftriaxone, cefotaxime, kanamycin, nalidixic acid, ciprofloxacin, tetracycline, sulfamethaxozale, chloramphenicol, and fosfomycin. The strain also exhibited a high MIC toward olaquindox (64 μg/ml). Conjugation experiments showed that the plasmid harbored by strain E80 was transferrable to the *E. coli* J53 recipient strain. The transconjugant also exhibited resistance toward most of the antibiotics tested including fosfomycin (MIC > 256 μg/ml), cephalosporins (>16 μg/ml), kanamycin (MIC = 128 μg/ml), chloramphenicol (MIC = 64 μg/ml), and nalidixic acid (MIC = 64 μg/ml). The MICs of olaquindox and ciprofloxacin were 64 μg/ml and 0.06 μg/ml, respectively. PCR screening showed that the transconjugant carried *oqxAB*, *fosA3*, and *bla*_CTX-M-1_ group resistance genes. The plasmid, designated pE80, was subsequently extracted from transconjugant and subjected to PacBio sequencing for further analysis.

**FIGURE 1:**
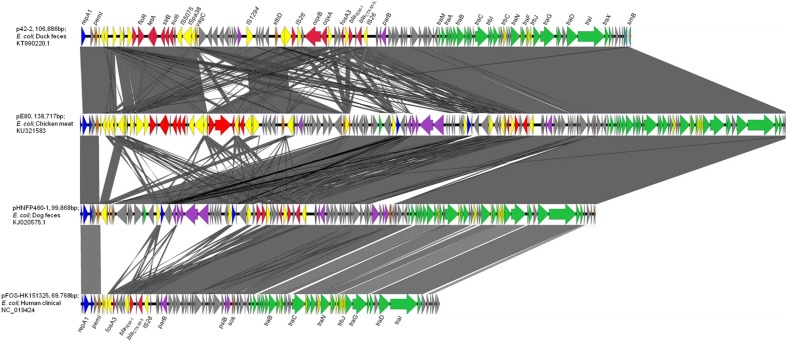
**Complete map and comparative analysis of pE80, p42-2, pHNFP460-1, and pFOS-HK151325.** Open reading frames (ORF) are depicted by arrows. Blue and purple arrows represent genes responsible for replication and maintenance; deep orange arrows depict genes responsible for plasmid stability; yellow and red arrows are mobile genetic elements and resistance genes, respectively; green and light green arrows represent *tra* and *trb* genes, respectively. The degree of genetic similarity between plasmids is depicted by the shaded area.

pE80 was found to be a 138,717 bp plasmid with a GC content of 51.87%, and belong to the IncFII-replicon type. The plasmid comprised 186 open reading frames, and exhibited the core features of the IncFII backbone including genes responsible for replication, stability, and conjugation. Multilocus sequence typing revealed that it belongs to the F33:A-:B- replicon. An additional IncN replicon gene was also identified. Interestingly, pE80 was found to comprise four plasmid addiction systems, *pemKI*, *stbDE*, *srnBC*, and *vagCD*, which may play a role in enhancing plasmid stability, although the *vagD* gene was interrupted by the insertion sequence IS*26*. Regarding transferability, 24 *tra* and 8 *trb* genes were found on this plasmid. BLASTN search revealed that pE80 is closely related to p42-2 (accession KT990220.1; 82% coverage; 99% identity), which was carried by a *E. coli* strain isolated from duck feces, and pHNFP460-1 (accession KJ020575.1; 71% coverage; 99% identity), which was carried by a *E. coli* strain isolated from dog feces in China (**Figure 1**). The plasmid also exhibits sequence similarity to other plasmids which harbor the *fosA3* and *bla*_CTX-M-1_ group elements, including pHN7A8 (accession JN232517.1, 52% coverage) which was recovered from an *E. coli* strain isolated from swine, and pFOS-HK151325 (accession JX627737.1, 46% coverage), which was harbored by a clinical *E. coli* isolate (He et al., 2013; Ho et al., 2013a) (**Figure 1**).

**FIGURE 2:**
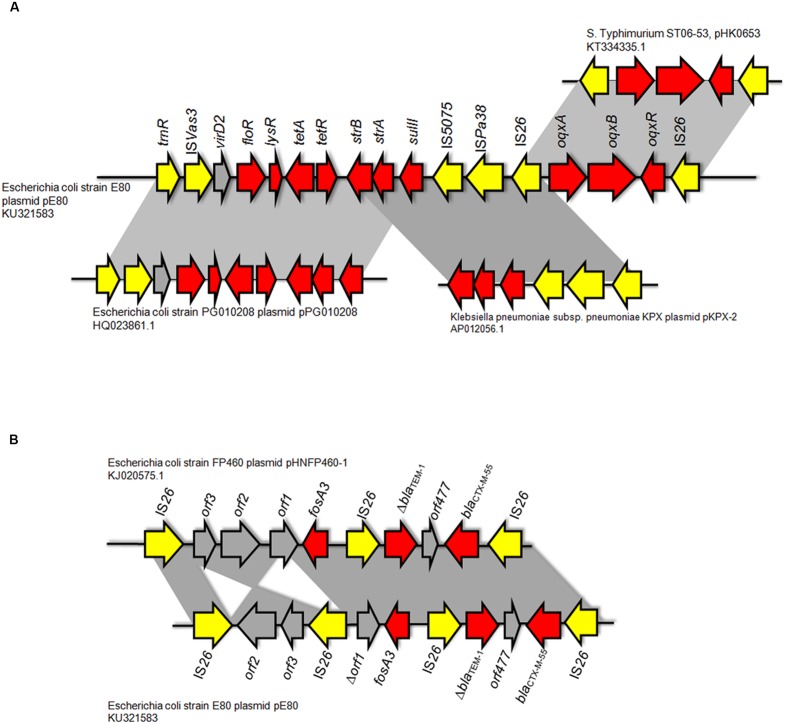
**Comparison between the genetic arrangement in the multidrug resistance region (MRR) in pE80 and other plasmids. (A)** Analysis of the first MRR in pE80. Specific regions in which genetic arrangements resemble those of other known plasmids are highlighted. **(B)** Comparison of the second MRR in pE80 harboring the *fosA3*, *bla*_TEM-1,_ and *bla*_CTX-M-55_ genes with that of the pHNFP460-1 plasmid. Genetic arrangements in the MRR of the two plasmids were highly similar except that the orientations of *orf2* and *orf3* were opposite to each other, and that an additional copy of IS*26* was found to be inserted into a site located between the truncated *orf1* and *orf3*, which is upstream of the *fosA3* gene.

Two Multidrug Resistance Regions (MRR) were identified in pE80. The first MRR covers the region of 9,750–34,791 bp, and was found to harbor multiple resistance genes, including *floR* (florfenicol/chloramphenicol resistance), *tetAR* (tetracycline eﬄux pump), *strB* and *strA* (streptomycin resistance), *sulII* (sulfonamide resistance), *oqxAB* (olaquindox/quinolone resistance), and *aph* (kanamycin resistance). Although a class I integrase gene was found, no corresponding class I integron gene cassette was detected. BLAST comparison was conducted and no identical MRR configuration could be identified. However, the genetic arrangements of MRR in pE80 was found to resemble those observed in several other plasmids. For instance, the *tnpR*-IS*Vas3*-*virD2*-*floR*-*tetAR*-*strBA*-*sulII* fragment in pE80 is frequently detectable in *bla*_CMY -2_-harboring IncA/C plasmids (Lee et al., 2015). Interestingly, the *strBA*-*sulII* region within this fragment, which was known to be linked to three transposase genes IS*5075*, IS*Pa38*, and IS*26*, were identical to the one which existed in another IncF plasmid, pKPX-2, which was recovered from a clinical *K. pneumoniae* isolate and known to comprise an *bla*_NDM-1_ element (Huang et al., 2013). The *oqxABR* locus beyond this fragment was flanked by two copies of IS*26*, and found to exist in the configuration of Tn*6010* in pHK0653 as well as in other *oqxAB*-harboring plasmids (**Figure 2A**). The second MRR was found to be located between 79,315 and 88,050 bp and comprise the resistance genes *fosA3*, *bla*_TEM-1,_ and *bla*_CTX-M-55_. This resistance region is flanked by the IS*26* transposase genes, a configuration commonly observed in other *fosA3*-bearing-IncFII plasmids (Yang et al., 2014). The configuration of this MRR, which comprises the genetic fragment of IS*26-orf2-orf3*-IS*26-Δorf1-fosA3*-IS*26-Δbla*_TEM-1_-*Δorf477-bla*_CTX-M-55_-IS*26*, exhibits slight variation to that of pHNFP460-1, and the *E. coli* plasmid pFOS-HK151325. In such fragment, the *orf2* and *orf3* downstream of *fosA3* are orientated in opposite directions, and that the *orf1* was truncated due to insertion of an additional copy of IS*26*; such genetic arrangement differs from that commonly observed in other plasmids (**Figure 2B**). Compared to another IncFII plasmid, p42-2, which harbored the same batch of resistance elements as that in pE80, sequence analysis revealed that the genetic arrangement varied significantly between two plasmids (Yang et al., 2016). The ∼26.5 kb IS*26*-flanked sequence comprising the *oqxAB* locus and some other accessory genes in pE80 was found to be oppositely oriented in p42-2. In addition, all the resistance elements in p42-2 are organized within a single MRR flanked by IS*1* sequences, whereas in pE80, a large fragment (∼27 kb) encoding accessory genes and hypothetical proteins (52,696–79,493 bp), also detectable in pHNFP460-1, was found inserted between *vagD* and the IS*26* linking *orf2*, adjacent to *fosA3* segment, resulting in formation of two different MRRs (**Figure 1**). Interestingly, the IS*26*-flanked MRR which contained *oqxAB*, together with the aforesaid ∼27 kb fragment encoding accessory genes, were both missing on pFOS-HK151325. Based on these data, we conclude that different transposition mechanisms were involved in capturing resistance elements in the chimeric plasmids of pE80 and p42-2, despite their structural similarity.

The PMQR gene *oqxAB* has been demonstrated to be associated with reduced fluoroquinolone susceptibility in *E. coli* and *Salmonella* spp., and was also found to be linked to nitrofurantoin resistance (Ho et al., 2016). While *oqxAB* is commonly carried by IncHI2 and IncFII plasmids (Liu et al., 2013; Li et al., 2014), *bla*_CTX-M-1_ group elements and *fosA3*, as well as the aminoglycoside resistance gene *rmtB*, have been frequently detected on IncFII plasmids carried by *E. coli* isolates of various origins (Pan et al., 2014; Alrowais et al., 2015). The co-existence of *oqxAB*, *bla*_CTX-M-55_ and *fosA3*, together with other resistance genes in pE80, resembles a similar but distinct genetic arrangement of the previously described plasmids p42-2 and pHNFP460-1. The presence of various MDR regions in pE80 and other plasmids provided evidence that the transmission and reassembly of different multidrug-resistant mobile elements resulted in formation of multidrug-resistance plasmids. Considering the fact that pE80, p42-2, and pHNFP460-1 were harbored by *E coli* strains isolated from chicken meat, duck feces, and dog fecal material, respectively, it is postulated that similar plasmids may have been widely disseminated in different environment settings in China. In order to determine the dissemination of pE80-like plasmid, PCR screening using various primer sets targeting to different regions of pE80 was performed on 190 *E. coli* food isolates collected during 2010–2012. Ninety-two out of 190 isolates tested contained IncFII type plasmids but they showed varied positive patterns in PCR screening and none of them were of high similarity to pE80 (Data not shown). The data revealed that even recovery of these IncFII multi-resistance plasmids has been described, its dissemination within environmental setting is not common. Nevertheless, the potential spread of plasmids similar to pE80 is of significant concern, as fosfomycin, third generation cephalosporins, fluoroquinolones, and nitrofurantoin are important agents in infection management. Co-localization of *fosA3*, *oqxAB*, and *bla*_CTX-M-55_ on multi-resistance plasmids and the subsequent transfer of these genetic elements to clinical strains are expected to confer a wide spectrum of antimicrobial resistance phenotypes, and may significantly compromise the effectiveness of current infection control measures.

### NUCLEOTIDE SEQUENCE ACCESSION NUMBER

The complete sequence of pE80 has been deposited in GenBank with an accession number KU321583.

## Author Contributions

MW designed and performed the experiment, analyzed the data, and wrote the manuscript. MX designed and performed the experiment, analyzed the data. LX and YZ analyzed the data. DL and RL performed the experiment. EC and SC analyzed the data and wrote the manuscript.

## Conflict of Interest Statement

The authors declare that the research was conducted in the absence of any commercial or financial relationships that could be construed as a potential conflict of interest.
